# Supplementary material for: Urinary microRNAs as Prognostic Biomarkers for Predicting the Efficacy of Immune Checkpoint Inhibitors in Patients with Urothelial Carcinoma
Source: Cancers (Basel). 2025 Aug 13;17(16):2640. doi: 10.3390/cancers17162640 (PMC12385101; doi:10.3390/cancers17162640)
Supplement: Supplementary file 1 [file cancers-17-02640-s001.zip › cancers-3747458-supplementary.pdf]

cancers

MDPI

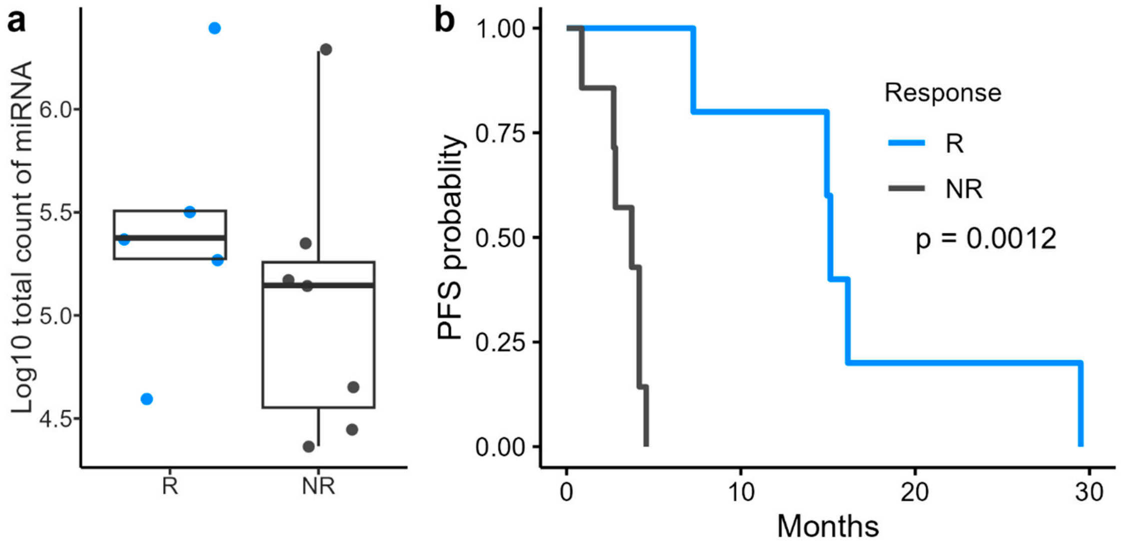

Figure S1. a The difference of the number of microRNA in urine sample between the responder and nonresponder groups. **Figure S1b** Progression-free survival of patients in the responder and nonresponder groups.

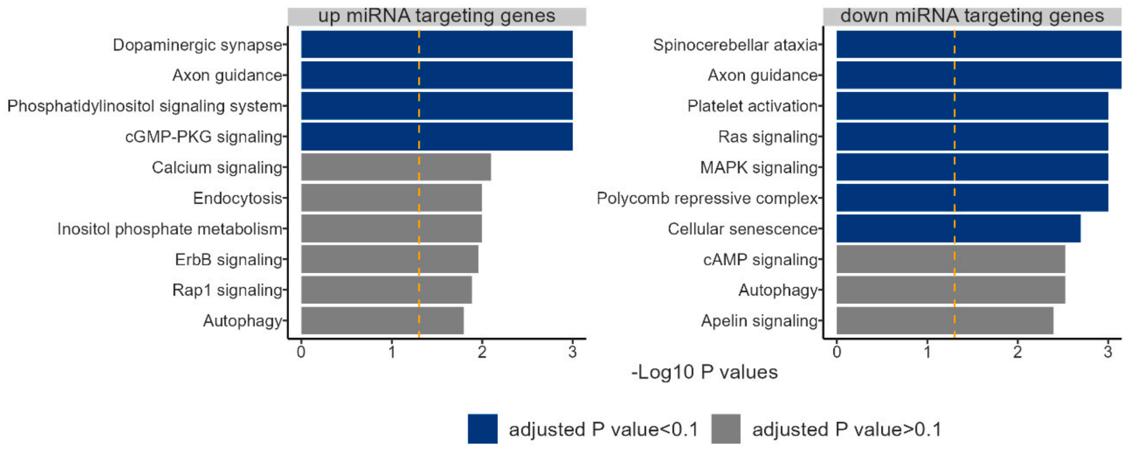

Figure S2. Pathway analysis of the target mRNAs of microRNA upregulated in responders identified four enriched pathways, whereas analysis of those upregulated in nonresponders identified seven enriched pathways.

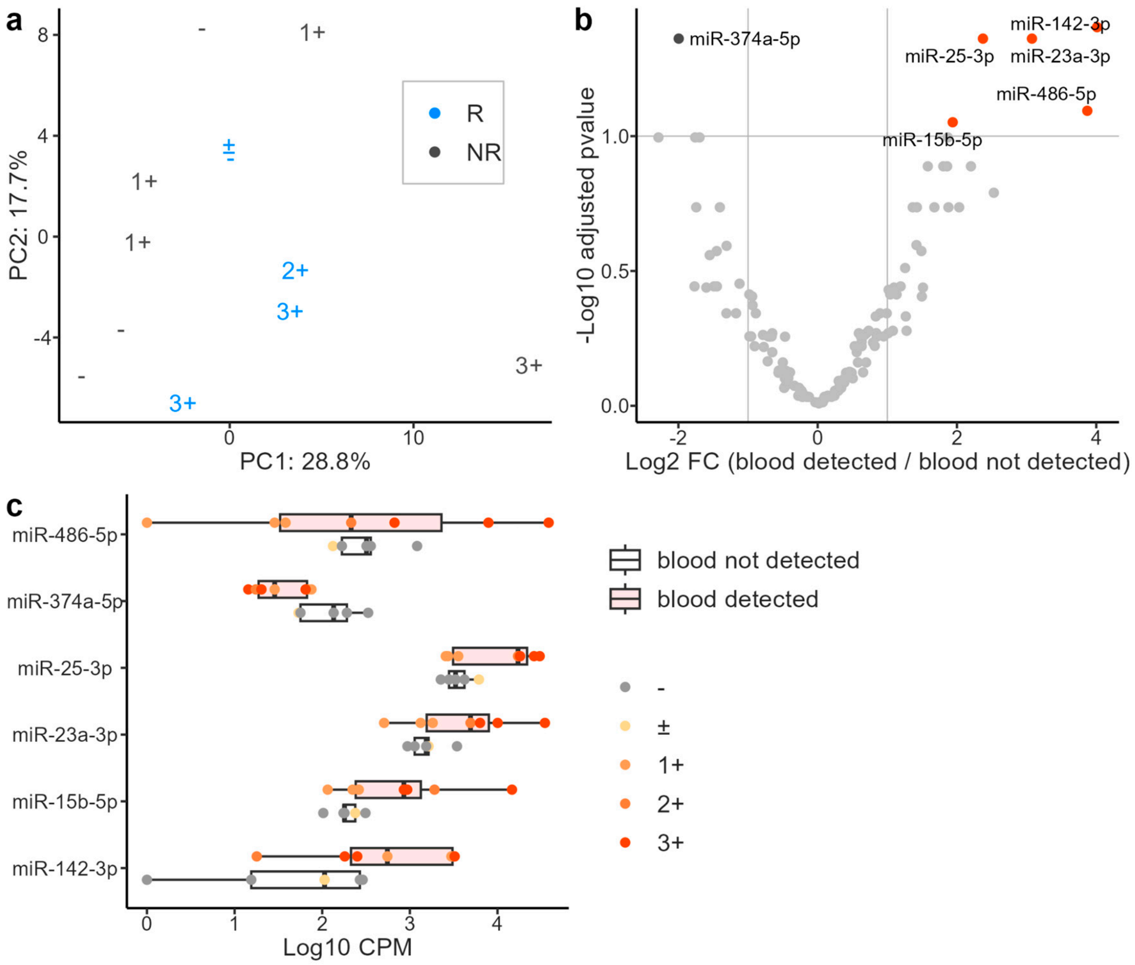

Figure S3. The effect of blood contamination for microRNA profiles in urine samples. (a) Principal component analysis between R and NR. Colors indicate response groups (R or NR), and labels (-, ±, 1+, 2+, and 3+) indicate urinary occult blood detection levels. (b) Differential expression analysis between blood detected (1+, 2+, and 3+) and blood not detected (- and ±) samples. (c) Expression levels of differentially expressed miRNA between blood detected and not detected samples.

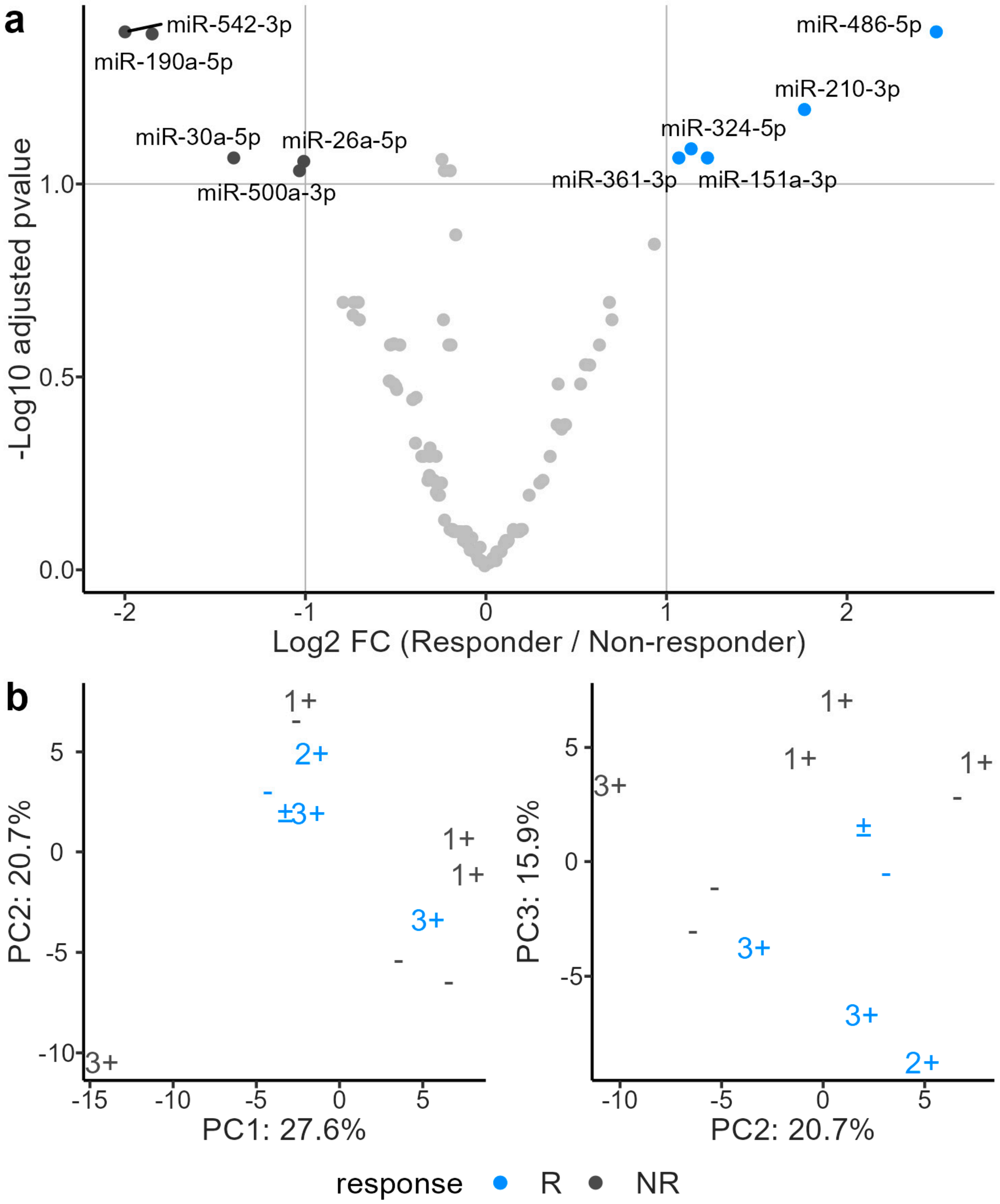

Figure S4. Adjusted miRNA profile by urinary occult blood. (a) Differential expression analysis adjusted for the presence of occult blood. (b) Principal component analysis of urinary miRNA profiles adjusted for occult blood.
